# Generative Modeling and Multispectral Imaging for Eye Fundus Classification

**DOI:** 10.3390/medsci14040488

**Published:** 2026-08-16

**Authors:** Francisco J. Burgos-Fernández, Buntheng Ly, Marina Bou-Marin, Fernando Díaz-Doutón, Jaume Pujol, Maxime Sermesant, Meritxell Vilaseca

**Affiliations:** 1 Centre for Sensors, Instruments and Systems Development, Universitat Politècnica de Catalunya, Rambla Sant Nebridi 10, 08222 Terrassa, Spain; marina.bou@upc.edu (M.B.-M.); fernando.diaz-douton@upc.edu (F.D.-D.); jaume.pujol@upc.edu (J.P.); meritxell.vilaseca@upc.edu (M.V.); 2 Institut Hospitalo-Universitaire Liryc, Avenue du Haut Lévêque, 33604 Pessac, France; buntheng.ly@inria.fr (B.L.); maxime.sermesant@inria.fr (M.S.); 3 Centre Inria d’Université Côte d’Azur, 2004 Route des Lucioles, 06902 Sophia Antipolis, France

**Keywords:** eye fundus, retina, choroid, multispectral imaging, variational autoencoder

## Abstract

**Background**: The early diagnosis of eye fundus pathologies is crucial, as they may go unnoticed until reaching advanced stages. To offer an improved screening methodology for this purpose, the effectiveness of a conditional variational autoencoder (CVAE) based on multispectral (MS) imaging and operating from the visible to the near-infrared range (416–1213 nm) has been assessed. **Methods**: A total of 2040 images from 102 patients (66 females, 36 males; aged 19–91 years) were acquired with an MS fundus camera to feed a fine-tuned CVAE for classifying eye fundus as healthy or diseased. The performance of the neural network was assessed for different image resolutions and spectral ranges. **Results**: The proposed deep generative model showed excellent results, reaching 100% of accuracy, sensitivity and specificity for the set of MS images from 416 nm to 955 nm at maximum resolution (1757 × 1757 pixels). Other instances with different image resolutions and spectral ranges led to good classifications (accuracy between 96% and 98%, sensitivity between 92% and 98%, and specificity between 97% and 100%). The CVAE exhibited robust performance with convergence of the accuracy and loss through the different epochs for training and validation in all instances. **Conclusions**: This study proves that a CVAE approach based on MS imaging is a highly effective tool for diagnosing eye fundus conditions and could potentially serve as a valuable clinical support tool for screening. The approach performs remarkably well when high spatial resolution MS images ranging from 416 nm to 955 nm are used. This underscores the importance of combining spatial and spectral information, particularly of wavelengths beyond the visible range.

## 1. Introduction

To prevent vision loss, many efforts have been directed toward assessing the posterior segment of the eye, that is, the fundus, which is crucial because many fundus pathologies do not manifest clinical signs until the disease reaches an advanced stage. This has fostered the major development of Deep Learning (DL) algorithms for computer-aided diagnosis in the field of ophthalmology [1,2,3]. Most DL techniques have been developed to aid in the diagnosis of age-related macular degeneration (AMD), diabetic retinopathy (DR), and glaucoma, the three main causes of severe vision loss affecting the eye fundus [4]. Typically, these algorithms rely on input data from color images acquired with traditional fundus cameras, which offer high spatial resolution. Access to several public datasets, some containing thousands of images, further facilitates their development and evaluation [5,6,7,8,9]. However, color images are inherently constrained by the phenomenon of metamerism, in which elements with spectrally different features can be perceived with the same color; thus, fundus abnormalities can sometimes be incorrectly identified as healthy structures.

Additionally, the huge variability in the features of fundus pathological signs hinders the grading of different disease stages. Currently, intensive work is being carried out in this field, yet substantial effort is required to develop robust models that can reliably assist ophthalmologists in grading fundus pathologies. These models demonstrate performance in terms of sensitivity and specificity close to 79% and 93% for AMD, 93% and 94% for DR, and 92% and 91% for glaucoma [2,3]. However, screening algorithms, which differentiate between a healthy and diseased fundus, have reported superior results in detecting fundus anomalies even though they may not specifically identify or grade the alteration. This is especially relevant because, in many cases, the signs of a disease are extremely subtle at the very first stages and are difficult to identify, even for experienced physicians. Therefore, DL algorithms can make a substantial difference in early detection. Convolutional Neural Networks (CNNs) have been shown to be the most successful DL techniques in dealing with two-dimensional input data, such as color fundus images, achieving good performance for AMD, DR, and glaucoma screening and diagnosis.

AMD has been a primary target for DL algorithms due to its high prevalence in elderly populations. Various architectures, including complex data preprocessing networks, ensemble models, and generative adversarial networks (GANs), have achieved high performance for healthy versus AMD classification [10,11,12,13,14,15]. Similarly, DR screening and diagnosis have been widely addressed using transfer learning strategies, two-way DL architectures on blood-vessel maps, attention-aware CNNs, and variational autoencoders (VAEs) [16,17,18,19,20,21,22,23,24,25]. Glaucoma detection has also been extensively studied through multilevel CNNs, optic disk and blood vessel segmentation models, and autoencoder architectures [26,27,28,29,30,31,32,33].

Although these DL models applied to conventional color fundus images have shown satisfactory performance, they remain fundamentally limited by metamerism. Therefore, some authors have directed their efforts toward DL techniques for the classification of multispectral (MS) fundus images. MS fundus cameras enhance the extraction of spectral information from the fundus by utilizing more than three spectral bands that are typically found in color images. This technology effectively combines the benefits of spectroscopy with imaging, thereby providing comprehensive spectral and spatial information on retinal landmarks [34,35,36].

Concerning the relevance of the amount of information for the DL mechanism, spectral imaging data have a remarkable benefit: they intrinsically contain real data augmentation because every fundus structure possesses its own variant at different spectral ranges. Che et al. [37] proposed an autoencoder for image registration of MS fundus images (550–850 nm), but not for classification. A second study [38] targeting blood vessels showed an improved version of this network. Following the same approach, another group developed a DL approach for DR lesion segmentation that is based on local binary patterns generated from MS images (550–850 nm) and fed them into a generalized low-rank approximation of matrices, although DR diagnosis was not implemented [39]. Finally, they merged image segmentation and registration in a study by Jiang et al. [40]. Specifically, they used an MS fundus camera that does not capture information from short visible (VIS) wavelengths (<550 nm), which are crucial for studying the nerve fiber layer and fovea [41]. Therefore, the diagnosis of fundus pathologies using MS imaging and DL techniques remains a challenge.

To this end, we propose a conditional variational autoencoder (CVAE) as a diagnostic tool for fundus pathology screening based on MS imaging operating from the VIS to the near-infrared (NIR) range (416–1213 nm). Notably, the inclusion of NIR wavelengths in addition to VIS light enables the imaging of deeper layers such as the choroid, which are typically inaccessible using traditional color fundus cameras. This approach combines MS technology and a deep generative model to classify the analyzed fundus, which encompasses a wide range of pathologies such as AMD, glaucoma, and vitreous detachment into either healthy or diseased categories. The proposed CVAE showed outstanding performance.

## 2. Materials and Methods

### 2.1. Subjects

The eyes of 49 patients with no ocular disease (control group) and 53 patients exhibiting any alteration in the fundus were studied (disease group). Trials were conducted at the Instituto de Microcirugía Ocular (IMO-Miranza Group, Barcelona, Spain) and the Vision University Centre (CUV) of the Universitat Politècnica de Catalunya (Terrassa, Spain). The Declaration of Helsinki tenets of 1975 (revised in Tokyo in 2004) were followed throughout this study. Ethical committee approval was obtained (code: CODIMO 190129_138) and all patients provided written informed consent before undergoing any examination. Table 1 shows patient data following the established clinical requirements.

General inclusion criteria were subjective spherical refraction between ±15D and astigmatism ≤ 2D, both limited by the operation range of the MS fundus camera used. For the control group, the inclusion criteria were as follows: best-corrected visual acuity (BCVA) equal to or higher than 0.9 in decimal units (20/22.2 Snellen), intraocular pressure (IOP) ≤ 21 mmHg, normal fundus, and no history of any other ocular condition. For the diseased group, fundus pathologies such as DR, AMD, or glaucoma were included. The exclusion criteria included a diagnosis of any other ocular or systemic disease affecting the eye differently from previous diseases, particularly those that alter the transparency of the ocular media. To balance both datasets, 68 healthy and 68 diseased eyes were included in the analysis.

### 2.2. Multispectral Fundus Imaging

**Figure 1:**
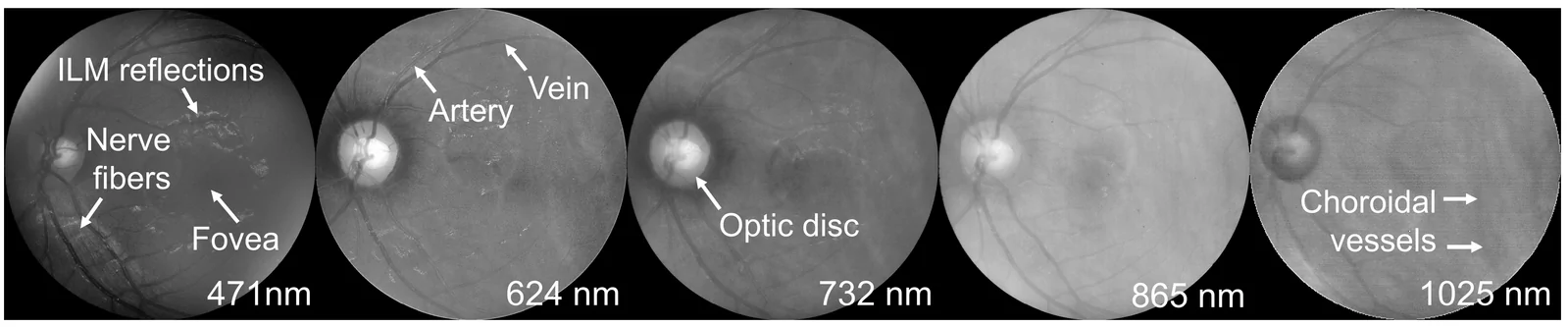
Representative VIS and NIR fundus images of a healthy eye captured with the purpose-built MS fundus camera. Key anatomical structures are labeled: nerve fibers, fovea, retinal artery and vein, optic disk and choroidal vessels. An additional label at 471 nm indicates the physiological specular reflections of the inner limiting membrane (ILM), which are originated from Müller cell footplates. They are considered a normal clinical feature, more prominent in young subjects due to higher ocular media transparency.

A purpose-built MS fundus camera was used to acquire fundus images in the VIS and NIR ranges, the last being relatively unexplored for this application as formerly explained [36]. Its main components consist of a CMOS camera (400–1000 nm), an InGaAs camera (950–1300 nm), and a LED-based light source with 15 spectral bands (416, 450, 471, 494, 524, 595, 598, 624, 660, 732, 865, 955, 1025, 1096, and 1213 nm); despite the number of spectral bands, the measurement only lasts 612 ms, thereby minimizing image artifacts and patient’s discomfort. As shown in Table 1, the size of the dataset (136 eyes) was considered relatively small for the DL model. However, 15 images with different spectral information were acquired for each eye, thereby providing a substantial amount of valuable input data for the algorithm (136 eyes × 15 images = 2040 images). Figure 1 shows some MS images acquired using the developed device.

### 2.3. Image Dataset

For each eye, each set of fundus images consisted of two subsets: 12 images captured using a CMOS camera (1757 × 1757 pixels) and 3 images captured using an InGaAs camera (386 × 386 pixels). Given the significant difference in the image resolution between the two cameras, the CVAE was modified and tested under various scenarios to determine the optimal configuration. To study the influence of different image resolutions, images were resized to 1757 × 1757 pixels or 386 × 386 pixels, depending on their original resolution and, if required, for the different instances. If a subset of images did not present the required image resolution for an instance, it was downscaled or upscaled to satisfy this requirement. Additionally, the amount of MS data used to train the model was analyzed to quantify the necessity of using extended NIR information to classify fundus images using a CVAE. Three spectral ranges were considered: VIS, 416–732 nm (CMOS camera); VIS-NIR, 416–955 nm (CMOS camera); and VIS-exNIR, 416–1213 nm (CMOS and InGaAs cameras). Training only with the three images acquired with the InGaAs camera (1025–1213 nm) was discarded because they were highly affected by water absorption by the ocular media. This resulted in a loss of light reflected by the fundus, thus producing lower-quality images compared to those from a CMOS camera. Table 2 summarizes the image resolutions and spectral ranges used as input data in the generative model.

### 2.4. Conditional Variational Autoencoder

**Figure 2:**
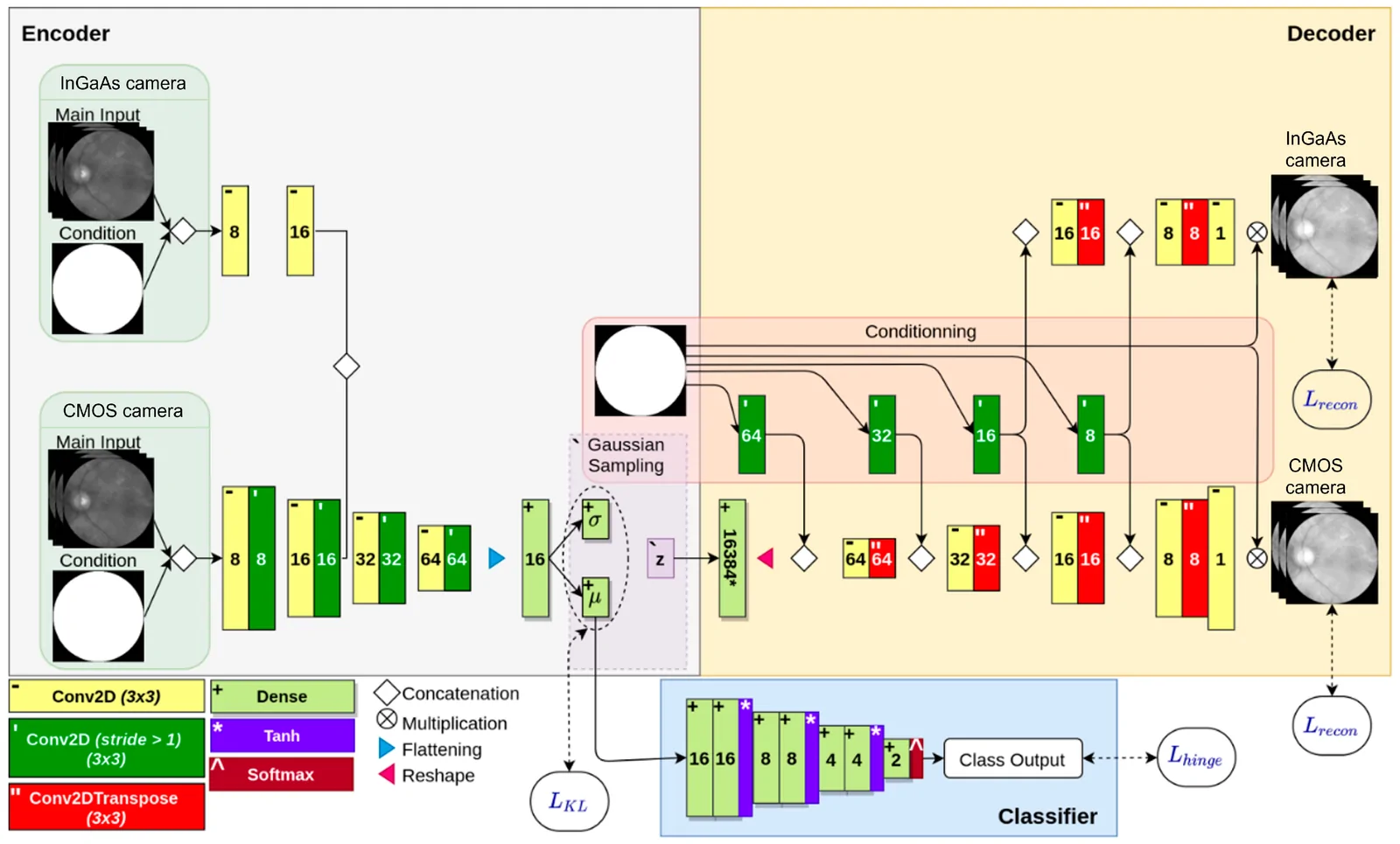
CVAE architecture when the whole set of 15 images is used. The color or the symbol in the bottom-left corner indicates the layer types. The number in each layer refers to the number of output filters or units. L_KL_, L_Hinge_, and L_recon_ refer to the Kullback–Leibler divergence loss, hinge loss, and reconstruction loss, respectively. Figure adapted with permission from Ly et al. (2021) [42] © Springer Nature.

A CVAE, originally designed for sustained ventricular arrhythmia prediction [42], was fine-tuned to assess the eye fundus using MS imaging. Figure 2 shows the network architecture of the CVAE. It consists of an encoder, a decoder branch, and a classifier model. In the encoder, the fundus images (‘Main input’ in Figure 2) are combined with a mask (‘Condition’ in Figure 2) by a concatenation layer, followed by a series of two-dimensional convolution layers to generate a low dimensional space that feeds the classifier. Using the low-dimensional features as inputs, the decoder applies regular and transposed convolution layers to artificially reconstruct the original eye fundus images to concatenate them with the corresponding masks. The main advantage of the autoencoder architecture is the comparison between the encoder–decoder outputs and the original data because the resulting error is backpropagated through the network at each iteration to improve its performance, while also optimizing the classification performance. This enables the generation of a low-dimensional representation of the data that discriminates between healthy and diseased fundus images. Five randomly sampled sets were evaluated to validate the efficiency of the network. To account for potential inter-eye dependency within the same subject and prevent data leakage, dataset partitioning was performed strictly at the patient level. Thus, all eyes belonging to the same subject were assigned exclusively to either the training/validation subset (80% subjects = 108 eyes) or the test subset (20% data subjects = 28 eyes), ensuring that the test set consisted entirely of unseen subjects. Due to the different camera resolutions, the encoder and decoder were accordingly modified to be able to work with images from different devices in a multimodal arrangement (see ‘CMOS camera’ and ‘InGaAs camera’ in Figure 2). The Adam optimizer was used with an initial learning rate of 1× 10^−4^, which was reduced by half after five epochs with no validation improvement. A total of 100 epochs were used for the training. A computer with an AMD Ryzen 9 3950X 16-Core processor (Advanced Micro Devices, Inc., Santa Clara, CA, USA), 64GB RAM, and a Quadro RTX 4000 GPU was used. Python 3.8.12, and TensorFlow GPU 2.4 release together with CUDA v.10.8 libraries were used to perform training on the GPU; Keras 2.4.3 was used as an API.

### 2.5. Model Evaluation

The accuracy, sensitivity, specificity, and loss were selected to quantify the effectiveness of the model over the test subset. The accuracy is categorically based; therefore, it computes the percentage of predicted values that match the values of the one-hot ground truth labels. Sensitivity refers to the capability of the model to detect a diseased fundus, whereas specificity is intended for healthy fundus. The loss was computed as a combination of three types of loss: reconstruction loss, Kullback–Leibler divergence loss, and classification loss (hinge loss). The reconstruction loss was calculated using the weighted root mean squared error, which considers the output of the decoder, the input of the encoder, and the mask. The Kullback–Leibler divergence loss was obtained from latent and normal distributions. Finally, the hinge loss for classification was computed as the combined difference in each class between the ground truth and the model classification output. Further details on these metrics can be found in Ly et al. [42].

### 2.6. Technical Implementation and Execution Workflow

To ensure technical reproducibility and optimal execution of the proposed CVAE model, specific preprocessing and quality assurance protocols were established. Since MS image acquisition relied on two separate sensors, CMOS and InGaAs, spatial alignment between them was required. This image registration was performed manually using MATLAB R2018b (The MathWorks, Inc., Natick, MA, USA) by selecting 10 corresponding landmark control points on matching CMOS and InGaAs images for each eye. The resulting geometric transformation matrix was subsequently applied to align all spectral channels. To prevent degraded images from compromising model convergence, input data were subject to rigorous quality filtering, retaining only subjects who displayed high structural clarity and minimal noise across all evaluated spectral bands. To manage computational resource constraints and prevent GPU out-of-memory errors when training the network with these high-resolution inputs, the batch size was set to 1. Finally, the Kullback–Leibler divergence loss was implemented to ensure stability during CVAE optimization and avoid posterior collapse, which will cause the network to ignore the latent space and generate oversmoothed outputs.

## 3. Results

The performance of the CVAE in classifying fundus images as healthy or diseased is shown in Table 3. These results were calculated as the mean values for five randomly sampled sets, which yielded minimal loss when classifying the test subset.

**Figure 3:**
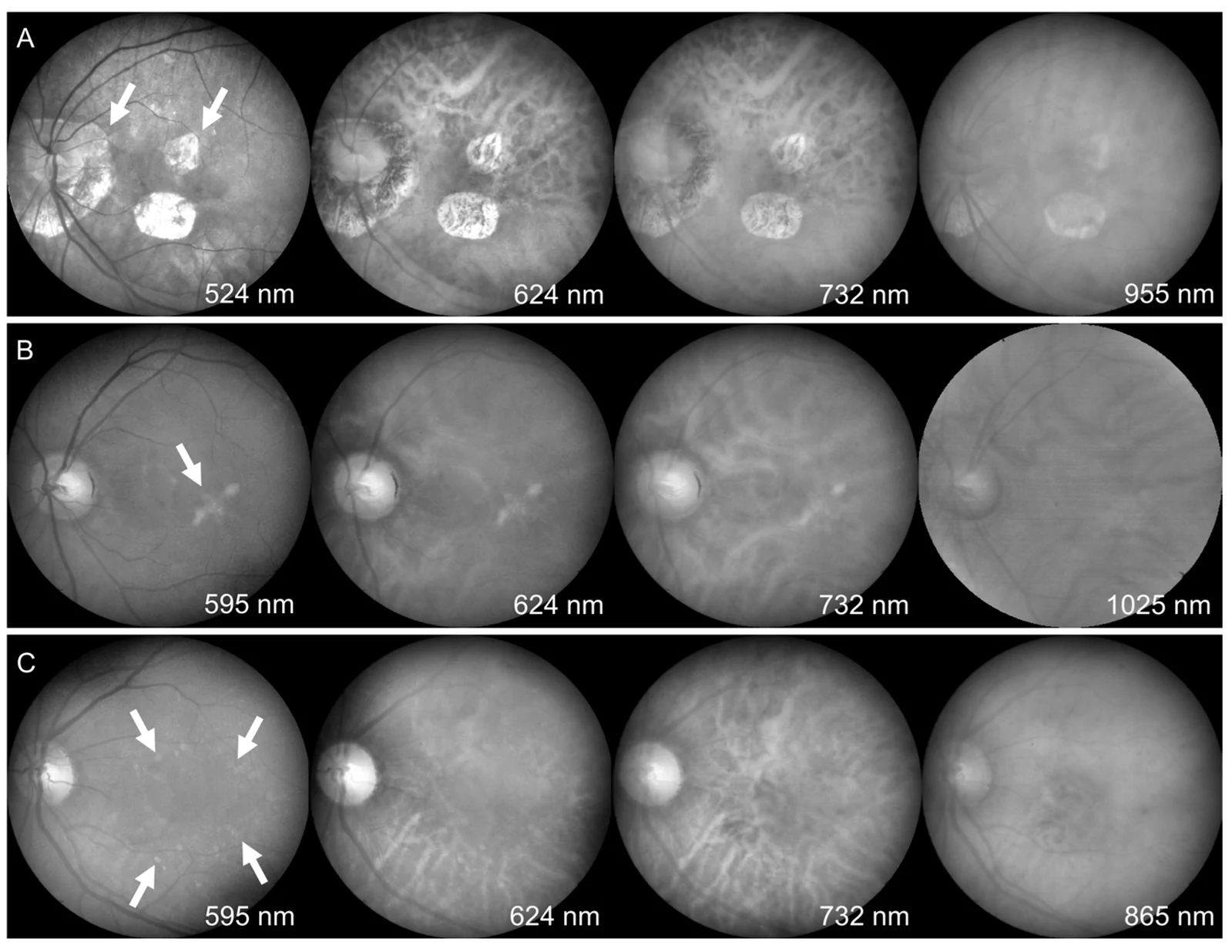
Representative VIS and NIR fundus images included in the study from eyes affected by different diseases and evidence of pathological structures: (**A**) Posterior vitreous detachment, high evidence; (**B**) butterfly macular dystrophy, medium evidence; (**C**) drusen (tiny white spots), low evidence. White arrows indicate the pathological structures.

The best results were obtained from the set of 12 images acquired with the CMOS camera at maximum resolution (1757 × 1757 pixels) in the spectral range of 416–955 nm, reaching a perfect score from the five randomly sampled test sets, that is, the accuracy, sensitivity, and specificity were 100% in all cases. This was followed by the model trained with images from the same spectral range and camera, but downscaled to 386 × 386 pixels. The third place was shared among three instances: two belonging to the whole set of 15 images, when (i) resized to 386 × 386 pixels, and (ii) using each set of images with their original resolution, and for the 10 images in the VIS range at their original resolution. At the last position, we found the set of 15 images resized to the maximum resolution, and that of 10 images in the VIS range downscaled to 386 × 386 pixels. In general, the performance of the CVAE was excellent for all instances, with accuracies, sensitivities, and specificities above 96%, 92%, and 97%, respectively. This is particularly relevant because fundus images include a wide spectrum of pathologies, ranging from severe and evident cases to early and subtle alterations caused by an extensive variety of conditions (Figure 3).

**Figure 4:**
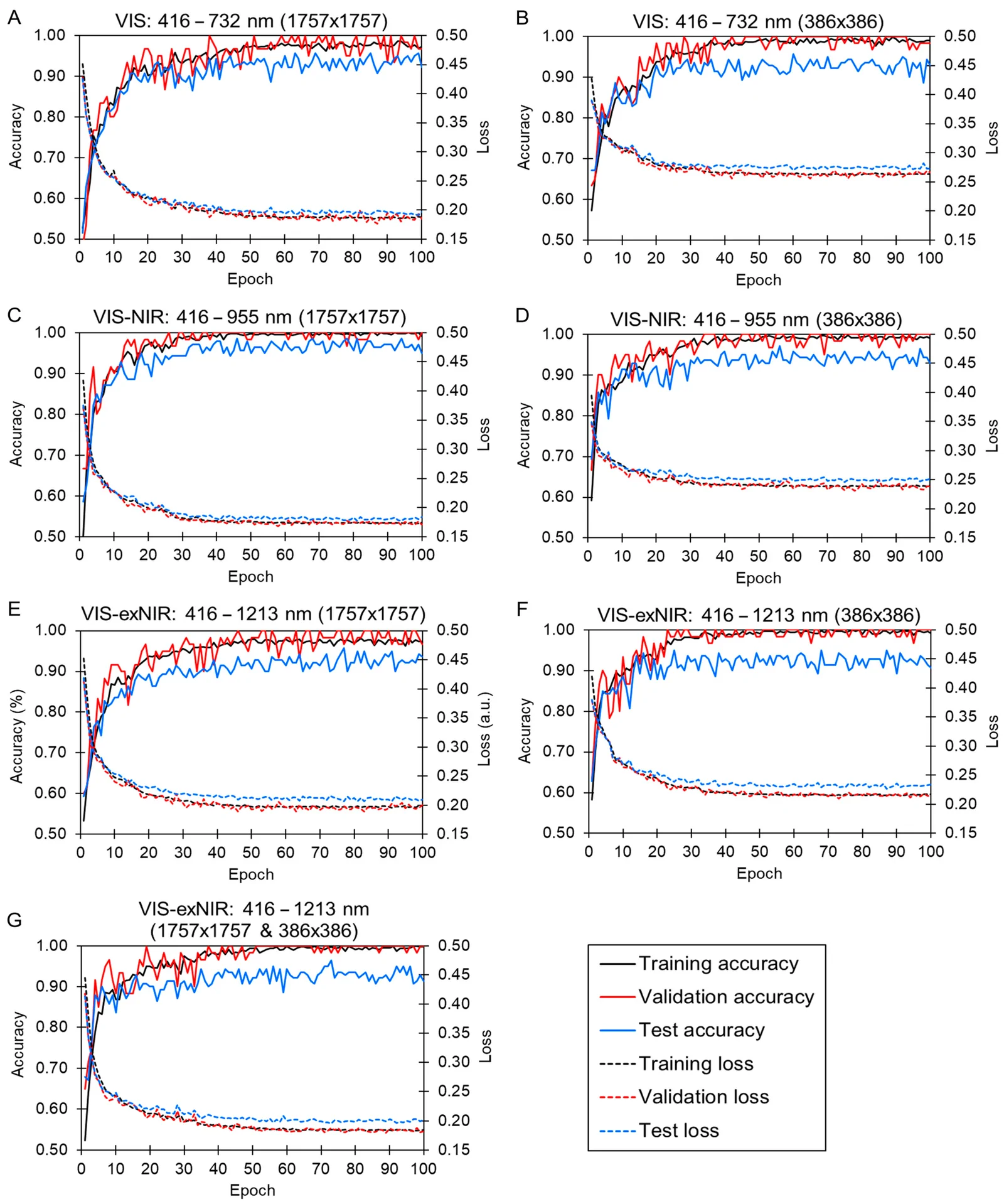
Mean values of the training, validation and test accuracy, and loss computed from the five randomly sampled sets and for the different instances of the CVAE as a function of spectral range and image resolution: VIS range from 416 nm to 732 nm for image resolutions of (**A**) 1757 × 1757 pixels, and (**B**) 386 × 386 pixels; VIS-NIR range from 416 nm to 955 nm for image resolutions of (**C**) 1757 × 1757 pixels, and (**D**) 386 × 386 pixels; VIS-exNIR range from 416 nm to 1213 nm for image resolutions of (**E**) 1757 × 1757 pixels, (**F**) 386 × 386 pixels, and (**G**) 1757 × 1757 pixels for the VIS-NIR range (416–955 nm), and 386 × 386 pixels for the exNIR range (1025–1213 nm), respectively.

The combined evolution and consistency of the training and validation accuracies and losses through the epochs for all model instances demonstrated that no overfitting was induced by the model at the stopping point of 100 epochs (Figure 4), and both parameters converged between the 40th and 50th epochs. The test accuracy and loss were also included for each instance.

The highest agreement between the training and validation accuracies was observed for the set of 12 images acquired with the CMOS camera (416–955 nm) at the maximum resolution (1757 × 1757 pixels) (Figure 4C), as evidenced by the 100% score in Table 3. As shown in Figure 4C, the test accuracy did not reach 100% because it occurred at a different epoch for each of the five randomly sampled sets. Therefore, it remained just below 100% when performing the mean at each epoch and differed slightly from the results in Table 3; the same occurred for the other instances. Major fluctuations during the validation stage occurred when working exclusively in the VIS range (Figure 4A) and when upscaling the images from the InGaAs camera to the maximum resolution, that is, 1757 × 1757 pixels (Figure 4E). The loss reached lower values (<0.20) when all images at the maximum and original resolutions were used (Figure 4A,C,E,G). This parameter was primarily affected by the classification and reconstruction losses when training with images at a minimum resolution (386 × 386 pixels).

## 4. Discussion

A CVAE network was trained on MS fundus images to enhance the discrimination between healthy and pathological eyes. Perfect separation between the two classes was achieved in one instance across all five randomly sampled sets. This revealed that training the network with maximum-resolution images (1757 × 1757 pixels) without resizing was crucial for precisely identifying pathological signs and differentiating them from healthy structures. Moreover, the most relevant spectral range was found to be between 416 and 955 nm, showing that spectral discrimination in the VIS range complemented with NIR information before strong water absorption (<1000 nm) is critical for fundus diagnosis. The fact that the second-best performance corresponded to the same spectral range further emphasizes its usefulness. To the best of our knowledge, this is the first time that a DL network has achieved 100% accuracy, sensitivity, and specificity for classifying healthy and pathological eye fundus. Therefore, working with high-resolution images and combining VIS and NIR data offers an advantage for the early diagnosis of fundus pathologies, significantly enhancing the ability to detect subtle alterations even at the initial stages. The other instances showed very precise classifications with accuracies between 96% and 98%, sensitivities between 92% and 98%, and specificities of up to 100%. However, the main requirement for physicians during diagnosis is to achieve 100% sensitivity to ensure that they can confidently detect any pathology and promptly proceed with treatment.

When compared with previous screening and diagnostic DL models using conventional color fundus images, the proposed CVAE approach demonstrates superior performance. Regarding AMD, Kunumpol et al. [10] built a complex data preprocessing model combining image contrast enhancement, feature extraction, and dimensionality reduction prior to an artificial neural network, reaching 98.6% sensitivity and 99.2% specificity. Bhuiyan et al. [11] assembled different architectures (Inception-V3 [12], Inception-Resnet-V2 [13], and Xception [14]) across various input sizes, with a logistic model tree achieving 98.9% sensitivity and 99.5% specificity. Additionally, generative models such as the semi-supervised GAN proposed by Smitha et al. [15] achieved around 90% accuracy for AMD detection. For DR, Aranha et al. [16] applied transfer learning using VGG16 [19] to low-quality fundus images, outperforming previous models [20,21] and obtaining 91.5% sensitivity and 84.9% specificity. Das et al. [17] developed a two-way classification architecture using squeeze, excitation, bottleneck, and pooling layers on pre-segmented blood-vessel maps, improving up to 99.6% sensitivity and 98.2% specificity compared to other schemes [22,23]. Maaliw et al. [18] combined extensive preprocessing with an attention-aware deep CNN (ResidualNet [24]) to avoid over-segmentation of fundus structures, reaching 99.4% sensitivity and 98.9% specificity. More recently, Sundar and Sumathy [25] used variational autoencoders (VAEs) for both detection and grading, reporting an average sensitivity of 87.0%. Similarly, glaucoma identification has been broadly addressed. Aamir et al. [26] designed a multilevel deep CNN to detect and grade glaucoma (Detection-Net and ClassificationNet) achieving 97.0% sensitivity and 99.0% specificity. Segmentation-based approaches have also shown notable success, as demonstrated by Islam et al. [27] who evaluated, among other models, EfficientNet-63 [28] on segmented optic cup and blood vessels, achieving 97.3% sensitivity and 100.0% specificity for discriminating between healthy and glaucomatous eyes; Natarajan et al. [29] combined a regularized U-Net [30] with cuckoo search optimization [31] to achieve 100.0% sensitivity and specificity on optic disk crops. Autoencoder (AE) architectures have been tested without prior segmentation of the optic disk, such as the two-layer cascaded AE by Raghavendra et al. [32], reaching 95.2% sensitivity; in contrast, Sathya and Balamurugan [33] applied AEs to previously segmented images of the optic disc using U-Net, with 97.01% sensitivity and 97.08% specificity. While these algorithms yield strong results for AMD, DR, and glaucoma, they operate on RGB data, which is inherently constrained by metamerism and subtle early-stage pathological variations.

In contrast, the CVAE model proposed in this work exploits the extensive spectral-spatial data provided by MS imaging. By combining VIS and NIR spectral information at maximum spatial resolution, our architecture achieves perfect classification performance while overcoming the fundamental limitations of metamerism and eliminating the need for complex, multi-step prior segmentation pipelines.

Computing time is another factor that is particularly relevant when addressing such large amounts of spectral and spatial information. Considering the computer used for one sample set, training the CVAE took less than 1 h for the minimum-resolution images (386 × 386 pixels), approximately 8 h for the sets preserving the original resolution, and 14 h for the entire set of MS images at maximum resolution (1757 × 1757 pixels), which could be significantly reduced by utilizing more powerful computing resources, such as a custom GPU server specifically designed for DL. Traditionally, DL architectures employ images of 512 × 512 pixels or smaller to optimize the computing time, thus taking advantage of datasets with hundreds or thousands of images. Based on the presented results, we conclude that using large images can be highly beneficial for training DL models with reduced datasets, particularly when precise spectral information is available. Despite the additional time required by the proposed approach during training, it is important to note that this time investment occurs only once and not in subsequent classifications. This makes the approach acceptable considering the excellent classification results achieved.

Furthermore, given the exceptionally wide diversity of fundus pathologies, developing an algorithm to identify and grade every specific disease requires large-scale clinical trials with vast patient cohorts, which was beyond the scope of this initial study. Consequently, to build a robust model, this work focused on a screening approach to differentiate between healthy and pathological fundus images, effectively consolidating the dataset into well-defined categories, thus maximizing the number of training samples per class.

Finally, the proposed CVAE was optimized for MS fundus cameras which are not yet as widely deployed in eye-care settings as other imaging modalities. Although this could be considered a limitation, the underlying generative framework can be adapted to other fundus imaging modalities, such as standard RGB imaging, or optical coherence tomography (OCT). Through suitable modification of key parameters, including spatial resolution, band dimensionality, and structural masking, the proposed framework offers the flexibility to learn from and classify alternative imaging data. Future iterations could incorporate transfer learning or domain adaptation techniques to allow the model to process other input data when MS data is unavailable, or explore lightweight network variants to reduce computational requirements without compromising classification accuracy.

## 5. Conclusions

The use of MS imaging and CVAE architecture for fundus diagnosis has proven to be an excellent alternative to color imaging for screening purposes. The different instances revealed that the most successful input data parameters were high image resolution (1757 × 1757 pixels) and the VIS-NIR spectral range (416–955 nm). This spectral range is significant because it avoids the use of costly InGaAs cameras, which typically provide a lower spatial resolution. Future studies will focus on expanding the dataset and adapting the network to grade the severity of various pathologies. This involves exploring attention maps and patch-based models. Furthermore, alternative modalities including RGB and OCT will be explored to construct a CVAE multimodal framework capable of exploiting complementary information across different imaging sources of the same fundus features.

## Figures and Tables

**Table 1:** Patient demographics, refraction and visual acuity (mean ± standard deviation [range]).

	Control Group	Diseased Group
Number of patients	49	53
Number of eyes	68	68 ^1^
Sex ^2^	29 F (59%); 20 M (41%)	37 F (70%); 16 M (30%)
Age (years)	41.82 ± 16.24 [20, 80]	67.04 ± 16.23 [19, 91]
Spherical refraction (D)	−0.01 ± 2.33 [−6.25, 8.00]	−0.32 ± 2.34 [−10.50, 4.50]
Astigmatism (D)	−0.80 ± 0.66 [−2.00, 0.00]	−0.73 ± 0.62 [−2.00, 0.00]
BCVA	1.05 ± 0.11 [0.90, 1.50]	0.64 ± 0.32 [0.01, 1.00]

^1^ Drusen, 10; wet AMD, 9; dry AMD, 9; glaucoma, 5; vitreous detachment, 5; alteration of the retinal pigmented epithelium, 4; retinal atrophy, 4; macular edema, 3; other fundus alterations (e.g., epiretinal membrane, macular hole, or scars), 19. ^2^ Biological attribute (F: female; M: male).

**Table 2:** Instances of the dataset considering image resolution and spectral range, indicating native resolution, downscaling from 1757 × 1757 pixels to 386 × 386 pixels, and upscaling from 386 × 386 pixels to 1757 × 1757 pixels.

Image Resolution Established for Each Instance (Pixels)	VIS: 416–732 nm	VIS-NIR: 416–955 nm	VIS-exNIR: 416–1213 nm
386 × 386	Downscaled	Downscaled	Downscaled ^1^
1757 × 1757	Native resolution	Native resolution	Upscaled ^2^
1757 × 1757 and 386 × 386 ^3^	-	-	Native resolution

^1^ Only the set of images provided by the CMOS camera (1757 × 1757 pixels) was downscaled. ^2^ Only the set of images provided by the InGaAs camera (386 × 386 pixels) was upscaled. ^3^ Neither the set of images provided by the CMOS camera (1757 × 1757 pixels) nor those provided by the InGaAs camera (386 × 386 pixels) were resized, thereby preserving the original image resolution of both sets.

**Table 3:** Performance of the different instances of the dataset considering image resolution and spectral range (mean ± standard deviation of accuracy [sensitivity, specificity]).

Image Resolution (Pixels)	VIS: 416–732 nm	VIS-NIR: 416–955 nm	VIS-exNIR: 416–1213 nm
386 × 386	96.43 ± 3.57 [92.86 ± 7.14, 100.00 ± 0.00]	97.86 ± 3.19 [97.14 ± 6.39, 98.57 ± 3.19]	97.14 ± 1.60 [95.71 ± 3.91, 98.57 ± 3.19]
1757 × 1757	97.14 ± 2.99 [97.14 ± 3.91, 97.14 ± 3.91]	100.00 ± 0.00 [100.00 ± 0.00, 100.00 ± 0.00]	96.43 ± 4.37 [95.71 ± 9.58, 97.14 ± 3.91]
1757 × 1757 & 386 × 386 *	-	-	97.14 ± 2.99 [94.29 ± 5.98, 100.00 ± 0.00]

* In this case, the images from both cameras were used simultaneously and at their original resolution; this approach was applied only for the VIS-exNIR instance.

## Data Availability

The data presented in this study are available on request from the corresponding author. The data are not publicly available due to privacy and ethical restrictions regarding participant confidentiality and the protection of clinical patient data.

## Abbreviations

The following abbreviations are used in this manuscript:

CVAE	Conditional variational autoencoder
MS	Multispectral
DL	Deep learning
AMD	Age-related macular degeneration
DR	Diabetic retinopathy
CNN	Convolutional neural network
VIS	Visible
NIR	Near-infrared
